# High Prevalence of Spirochetosis in Cholera Patients, Bangladesh

**DOI:** 10.3201/eid1504.081214

**Published:** 2009-04

**Authors:** Eric J. Nelson, Angela Tanudra, Ashrafuzzaman Chowdhury, Anne V. Kane, Firdausi Qadri, Stephen B. Calderwood, Jenifer Coburn, Andrew Camilli

**Affiliations:** Howard Hughes Medical Institute–Tufts University School of Medicine, Boston, Massachusetts, USA (E.J. Nelson, A. Camilli); Medical College of Wisconsin, Milwaukee, Wisconsin, USA (A. Tanudra, J. Coburn); Tufts Medical Center, Boston (A. Tanudra, A.V. Kane, J. Coburn); Jahangirnagar University, Dhaka, Bangladesh (A. Chowdhury); International Centre for Diarrhoeal Disease Research, Bangladesh, Dhaka (F. Qadri); Massachusetts General Hospital, Boston (S.B. Calderwood); Harvard Medical School, Boston (S.B. Calderwood)

**Keywords:** Spirochetosis, spirochete, cholera, Vibrio cholerae, Brachyspira, coinfection, secretory diarrhea, Bangladesh, dispatch

## Abstract

The microbes that accompany the etiologic agent of cholera, *Vibrio cholerae*, are only now being defined. In this study, spirochetes from the genus *Brachyspira* were identified at high titers in more than one third of cholera patients in Bangladesh. Spirochetosis should now be tracked in the setting of cholera outbreaks.

Cholera in humans results in profuse, watery diarrhea that can lead to severe dehydration and death (*1*). The infection begins with ingestion of *Vibrio cholerae* from contaminated water, after which expression of cholera toxin induces a fluid loss that may reach 1 liter/hour. Cholera is an underreported disease in the developing world, and the true incidence may reach 2 million cases/year (*2*).

Because anecdotal evidence has indicated frequent cholera and intestinal spirochetosis coinfection in Bangladesh, we studied both diseases in this study. Intestinal spirochetosis has negative effects on domestic swine and poultry industries (*3*). In humans, 1%–64% of colonic specimens demonstrate intestinal spirochetosis, with the highest prevalence in developing countries and immunocompromised populations (*4*). The etiologic agents of intestinal spirochetosis are members of the genus *Brachyspira* (formerly *Serpulina* and *Treponema*). *B*. *pilosicoli* isolated from humans cause disease in pigs and in chicken models of infection (*5*,*6*). *B*. *aalborgi* isolated from humans do not colonize animals (*7*). Surveillance of intestinal spirochetosis requires molecular tools because culture has limited sensitivity caused by the fastidious nature of *Brachyspira* spp. (*8*). Patients with symptomatic intestinal spirochetosis have chronic diarrhea or soft feces (*4*,*9*,*10*); these clinical signs may resolve with antimicrobial drug therapy (*9*). Histologic analysis of intestinal biopsy specimens showed densely packed spirochetes attached by 1 end to colonic epithelium, forming a false brush border (*11*). Invasion of colonic epithelial cells and bleeding may occur (*4*,*12*). Virulence mechanisms remain poorly understood. Research on a vaccine to protect pigs against intestinal spirochetosis has begun. However, there is no vaccine for protection against intestinal spirochetosis in humans.

Co-infection of cholera patients with additional pathogens has focused on enterotoxigenic *Escherichia coli* (ETEC); 13% of cholera patients in Bangladesh are co-infected with ETEC (*13*). The potential for pathogenic synergy between *V*. *cholerae* and other pathogens has been proposed but not investigated.

## The Study

We defined the frequent presence of spirochetes in feces of patients with cholera. A 3-step method was used to establish the distribution of spirochetes in rice-water stool: 1) the presence of spirochetes in rice-water stool was determined by using dark-field microscopy; 2) the diversity of *Brachyspira* spp. within a subset of these patients was quantified by 16S rDNA analysis; and 3) the genetic diversity within the most abundant species was determined by analysis of NADH oxidase (*nox*).

Rice-water stool samples were collected from symptomatic cholera patients (>15 years of age with no history of antimicrobial drug therapy) during the spring cholera outbreak of 2006 in Dhaka, Bangladesh, at the International Centre for Diarrheal Disease Research, Bangladesh, as part of a larger study (*14*). Samples were examined by dark-field microscopy for *V*. *cholerae* and other bacteria, and the presence of *V*. *cholerae*, lytic vibriophage, and ETEC was determined by using standard methods (*14*). Samples were preserved at 4°C in formalin, or cell pellets were resuspended in phosphate-buffered saline and 15% glycerol and stored at –80°C. Analysis showed that 36% (23/64) of samples that were positive for only *V*. *cholerae* also harbored spirochetes (Technical Appendix, panels A–F). Spirochetes were also found in 4/11 and 5/15 samples that harbored ETEC alone or both ETEC and *V*. *cholerae*, respectively. When we removed ETEC-positive samples as potential confounders, the presence of spirochetes was independent of lytic vibriophage in *V*. *cholerae*–positive stool samples (Technical Appendix, panel G), which is in contrast to the documented trend concerning non–*V*. *cholerae* bacteria in rice-water stool (*14*). The ratio of spirochetes to *V*. *cholerae* was ≈1 and independent of lytic vibriophage (Technical Appendix, panel H).

In the second step, 10 samples were chosen for molecular analysis on the basis of a large amount of spirochetes. Samples were heated (99°C for 10 min), and standard PCR for a diagnostic segment of the 16S rDNA gene (*15*) was performed by using genus *Brachyspira*–specific primers (5′-GTCTTAAGCATGCAAGTC-3′ and 5′-AACAGGCTAATAGGCCG-3′). Products were cloned and sequenced bidirectionally (GenBank accession nos. FJ599620–FJ599639). *B*. *pilosicoli* was the most common species, found in all 10 samples (Technical Appendix, panel I). *B*. *aalborgi* was the second most common species (7 samples). A second set of panspecific 16S rDNA degenerate primers (5′-GTTTGATYCTGGCTYAGARCKAACG-3′ and 5′-CCSSTACGGMTACCTTGTTACG-3′) confirmed the presence of *Brachyspira* spp. and suggested that spirochetes of other genera were not present. The added resolution of *nox* analysis also identified *B*. *hyodysenteriae* at lower abundance.

Culture, purification, and microscopy of *B*. *pilosicoli* from glycerol stocks was used to cross-validate the molecular approach. Standard fastidious anaerobe agar (FAA) supplemented with bovine blood (10%), spectinomycin (400 μg/mL), and polymyxin B (5 μg/mL) was determined empirically to be the optimal medium for isolation of spirochetes. Dilutions of glycerol stocks from each patient were plated on FAA agar; single colonies were best obtained with an FAA overlay. Plates were incubated for 21 days at 37°C in an atmosphere of 94% H_2_ and 6% CO_2_. Most colonies gave rise to sigmoidal spiral cell morphotypes similar to the morphotype of the American Type Culture Collection (ATCC) (Manassas, VA, USA) control strain for *B*. *pilosicoli*. Additional controls were ATCC *B*. *hyodysenteriae*, *Helicobacter pylori*, and *Borrelia burgdorferi*. Subsequent molecular analysis of patient isolates confirmed them as *B*. *pilosicoli*. Consistent with previous studies, *B*. *aalborgi* was not cultured from patient samples.

In the third step, 5 samples were analyzed by using *nox* sequence comparisons that yield higher phylogenetic resolution. DNA was extracted and a *nox* segment was amplified by using degenerate primers (5′-GCYGGHACATGGGCDGCAAAAAC-3′ and 5′-CAAATACRCAAATAGCRTTAG-3′). Products were cloned and sequenced bidirectionally (GenBank accession nos. FJ599589–FJ599619). *B*. *pilosicoli* was the most common *nox* sequence found. A phylogenetic tree (Figure) demonstrated that individual patients harbored clonal lines of *B*. *pilosicoli* (patients B, D, and E) or more diverse strains (patient A). Overall, *B*. *pilosicoli* strains found in cholera patients were extremely diverse relative to the known outgroup species, which indicates the potential for detection of new species related to intestinal spirochetosis.

**Figure:**
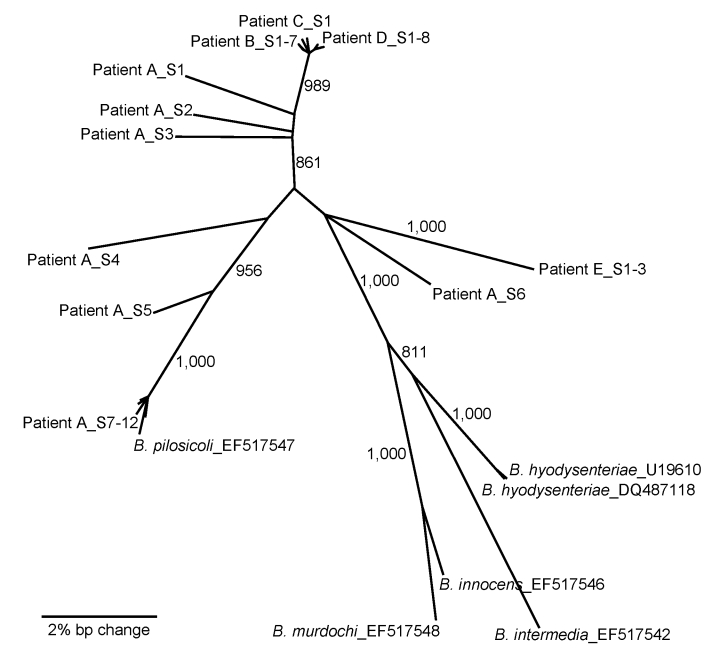
Neighbor-joining (NJ) phylogeny of NADH oxidase (*nox*) sequences of *Brachyspira pilosicoli* from 5 cholera patients (A–E). The *nox* sequences were PCR amplified, cloned, and sequenced from each patient (individual clones are appended _SX). Published sequences from known species are included for reference. NJ analysis was performed by using an NJ model and 1,000 bootstraps. Bootstrap values >800 are presented next to nodes. The scale bar indicates a 2% bp change (contiguous sequence ≈990 bp).

## Conclusions

It has been casually observed for a century that stool from cholera patients harbors spirochete-like bacteria. We now define the major agents present as *B*. *pilosicoli* and *B*. *aalborgi*. More than one third of the cholera patients had spirochetes in their stools at densities equal to those of *V*. *cholerae*. The pathophysiology of intestinal spirochetosis in this setting and its relevance to human health remain unknown. Epidemiologic analysis of intestinal spirochetosis has so far relied on retrospective studies of colonic tissue collected for reasons secondary to the disease (*4*). We recommend a community-based prospective study of stool samples from healthy persons and patients with diarrhea by using the techniques described herein; animal reservoirs should be identified as a potential point of control. In the context of *V*. *cholerae* infection, we hypothesize that spirochetes may be present before *V*. *cholerae* infection and exacerbate the already devastating clinical course of cholera.

## Supplementary Material

Technical Appendix Presence of spirochetes (Spiro) in cholera stools. Representative images of planktonic and aggregated spirochetes. *Vibrio cholerae* (VC) were labeled with a fluorescein isothiocyanate–conjugated monoclonal antibody (Ab) to O1 lipopolysaccharide (panels A and D), and nucleic acid was labeled with 4′,6-diamidino-2-phenylindole (DAPI) (panels B and E), which showed sigmoidal morphology with tapered ends (4–8 µm) indicative of spirochetes. The single curve or W shape is indicative of *Brachyspira pilosicoli*. Merged images (panels C and F) showed *V. cholerae* and spirochetes. The aggregate (panel F) was also stained for mucus (blue indicates wheat germ agglutinin lectin) and showed that only a small portion of the aggregate contained mucus. Panel G shows spirochetes counted by direct count microscopy in rice-water stool samples relative to the presence or absence of lytic vibriophage. Panel H shows the ratio of spirochetes to *V. cholerae* by direct count microscopy relative to the presence or absence of vibriophage. Panel I shows the percentage of spirochetes that were *B. pilosicoli* as determined by using partial sequence analysis of 16S rDNA. PCR amplified products from isolated DNA were cloned and sequenced. Those clones that did not harbor *B. pilosicoli* sequence harbored *B. aalborgi* sequence. NEG, negative; POS, positive; Tot, total. Horizontal bars are median values with interquartile range as whiskers; medians were not significantly different (p>0.05, by Mann-Whitney U test); Scale bars = 10 µm.
