# Supplementary material for: High Prevalence of Spirochetosis in Cholera Patients, Bangladesh
Source: Emerg Infect Dis. 2009 Apr;15(4):571–3. doi: 10.3201/eid1504.081214 (PMC2671413; doi:10.3201/eid1504.081214)
Supplement: Technical Appendix — Presence of spirochetes (Spiro) in cholera stools. Representative images of planktonic and aggregated spirochetes. Vibrio cholerae (VC) were labeled with a fluorescein isothiocyanate–conjugated monoclonal antibody (Ab) to O1 lipopolysaccharide (panels A and D), and nucleic acid was labeled with 4′,6-diamidino-2-phenylindole (DAPI) (panels B and E), which showed sigmoidal morphology with tapered ends (4–8 µm) indicative of spirochetes. The single curve or W shape is indicative of Brachyspira pilosicoli. Merged images (panels C and F) showed V. cholerae and spirochetes. The aggregate (panel F) was also stained for mucus (blue indicates wheat germ agglutinin lectin) and showed that only a small portion of the aggregate contained mucus. Panel G shows spirochetes counted by direct count microscopy in rice-water stool samples relative to the presence or absence of lytic vibriophage. Panel H shows the ratio of spirochetes to V. cholerae by direct count microscopy relative to the presence or absence of vibriophage. Panel I shows the percentage of spirochetes that were B. pilosicoli as determined by using partial sequence analysis of 16S rDNA. PCR amplified products from isolated DNA were cloned and sequenced. Those clones that did not harbor B. pilosicoli sequence harbored B. aalborgi sequence. NEG, negative; POS, positive; Tot, total. Horizontal bars are median values with interquartile range as whiskers; medians were not significantly different (p>0.05, by Mann-Whitney U test); Scale bars = 10 µm. [file 08-1214_Techapp-s1.pdf]

High Prevalence of Spirochetosis in Cholera Patients, Bangladesh

Technical Appendix

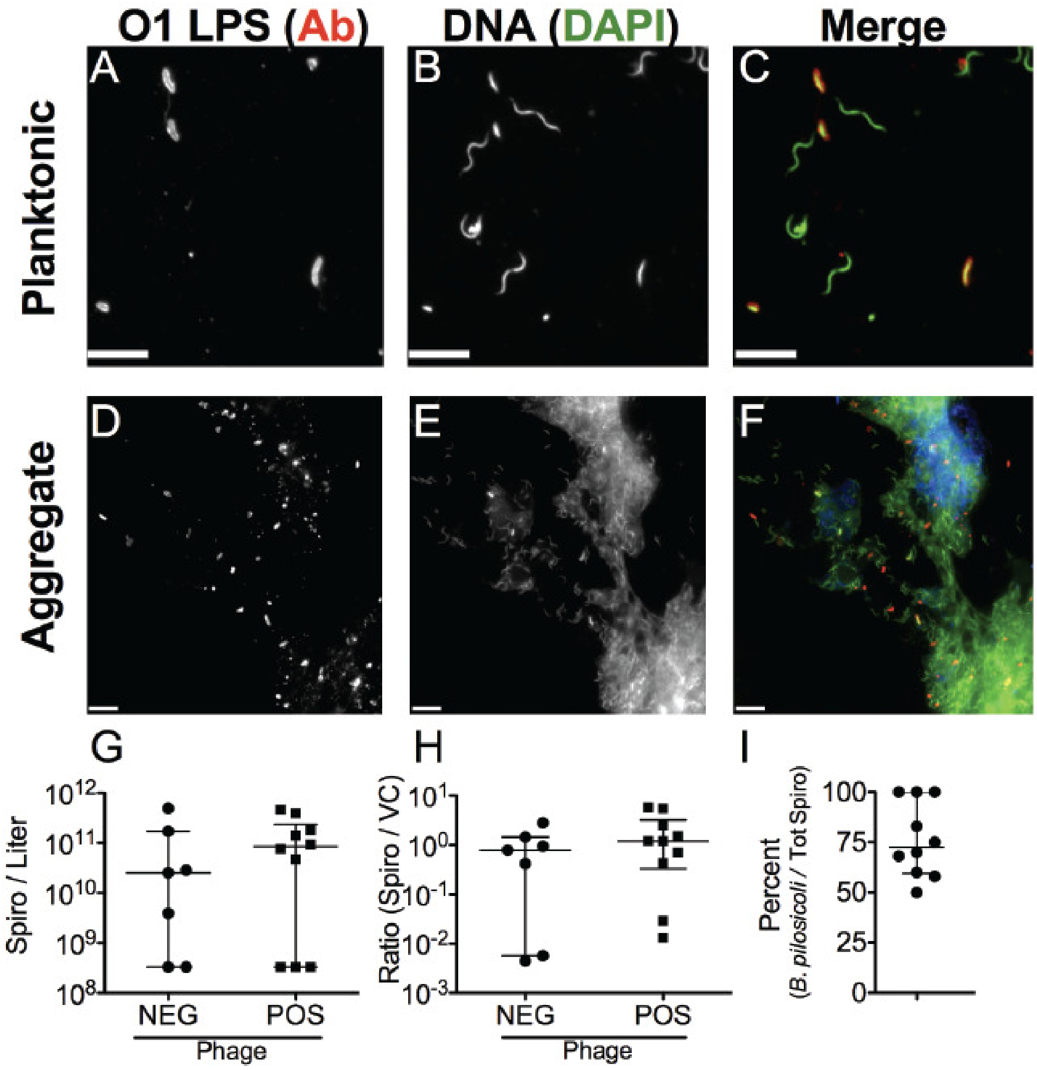

Presence of spirochetes (Spiro) in cholera stools. Representative images of planktonic and aggregated spirochetes. *Vibrio cholerae* (VC) were labeled with a fluorescein isothiocyanate–conjugated monoclonal antibody (Ab) to O1 lipopolysaccharide (panels A and D), and nucleic acid was labeled with 4′,6-diamidino-2-phenylindole (DAPI) (panels B and E), which showed sigmoidal morphology with tapered ends (4–8 µm) indicative of spirochetes. The single curve or W shape is indicative of *Brachyspira pilosicoli*. Merged images (panels C and F) showed *V. cholerae* and spirochetes. The aggregate (panel F) was also stained for mucus (blue indicates wheat germ agglutinin lectin) and showed that only a small

portion of the aggregate contained mucus. Panel G shows spirochetes counted by direct count microscopy in rice-water stool samples relative to the presence or absence of lytic vibriophage. Panel H shows the ratio of spirochetes to *V. cholerae* by direct count microscopy relative to the presence or absence of vibriophage. Panel I shows the percentage of spirochetes that were *B. pilosicoli* as determined by using partial sequence analysis of 16S rDNA. PCR amplified products from isolated DNA were cloned and sequenced. Those clones that did not harbor *B. pilosicoli* sequence harbored *B. aalborgi* sequence. NEG, negative; POS, positive; Tot, total. Horizontal bars are median values with interquartile range as whiskers; medians were not significantly different ($p>0.05$, by Mann-Whitney U test); Scale bars = 10 µm.
